# Suggested Indications for Enucleation of Solid Pseudopapillary Neoplasms in Pediatric Patients

**DOI:** 10.3389/fped.2019.00125

**Published:** 2019-04-03

**Authors:** Yu Jeong Cho, Jung-Man Namgoong, Dae Yeon Kim, Seong Chul Kim, Hyun Hee Kwon

**Affiliations:** Department of Pediatric Surgery, Asan Medical Center Children's Hospital, University of Ulsan College of Medicine, Seoul, South Korea

**Keywords:** solid pseudopapillary neoplasm, enucleation, pancreatic fistula, pancreas, diabetes mellitus

## Abstract

**Background:** Solid pseudopapillary neoplasms (SPNs) are rare, low-grade, malignant neoplasms that can occur in pediatric patients. Although complete resection of the tumor is the principle treatment, SPN enucleation (EN) has been reported to be effective in children. This study aimed to examine the feasibility and safety of EN by comparing it with conventional pancreatectomy (CP), and to present the indications for its use in pediatric patients.

**Methods:** We retrospectively reviewed the medical records of 66 patients who underwent surgery for SPN at our institution from October 1992 to April 2018. Surgical methods, postoperative complications, hospital stay, and recurrence were compared.

**Results:** Of the 66 patients, 15 (22.7%) were treated with EN and 51 (77.3%) were treated with CP. The mean duration of EN operation was 262 min (±145 min) and of CP was 345 min (±195 min). There was no statistically significant difference between the two methods (*P* = 0.13). To objectively compare the mass size between patients, we introduced a tumor size/intraperitoneal width ratio, which also revealed no significant difference between the 2 surgery groups (*P* = 0.21). The EN group had one case of recurrence at the resection site. The complications observed were fluid collection, splenic infarctions, hematomas, pancreatic fistulas, portal vein thromboses, and chylous drainage, among which pancreatic fistulas were the most frequent followed by moderate-severe fistulas in the EN group (*P* < 0.001). The mean postoperative fasting time (EN 17.0 ± 8.7 days vs. CP 5.1 ± 3.3 days, *P* < 0.001) and mean hospital stay (EN 23.4 ± 10.0 days vs. CP 13.2 ± 6.5 days, *P* = 0.002) showed statistically significant differences.

**Conclusion:** Compared with CP treatment, EN of SPNs in children has the disadvantages of prolonged fasting times and hospital stays to recover from moderate pancreatic fistulas. However, if appropriate indications are applied, EN can be considered a safe and effective surgical procedure for children.

## Introduction

Solid pseudopapillary neoplasm (SPN) is a rare pancreatic disorder that was first introduced by Virginia Frantz in 1959 (1, 2). This low-grade malignant neoplasm of the pancreas accounts for ~1–3% of all cases of pancreatic neoplasms, occurring mostly in young women (3). However, the development of imaging techniques and the broader use of cross-sectional imaging have led to better recognition of this disease in the past few decades, resulting in its increased detection and diagnosis (3, 4).

Although complete resection of the tumor by current conventional pancreatectomy (CP) is the main treatment of choice, there are considerations of its use in children despite its safe implementation. This is due mainly to the fact that the procedure still has a high morbidity rate (40–60%) and causes loss of endocrine or exocrine function because of a significant loss of normal tissue (3, 5–7). The incidence of diabetes mellitus (DM) following pancreaticoduodenectomy varies from 15 to 40%, and increases to 72% in the case of distal pancreatectomy (8).

Therefore, enucleation (EN), which is a less aggressive treatment that preserves the normal parenchyma, has been suggested for the surgical management of low-risk malignancies, with a variety of studies reporting its efficiency and safety (Table 1) (3, 7, 10–12). Wang et al. compared 31 patients who had undergone EN with 70 patients who had undergone CP, showing an improved outcome in the EN group with a lower rate of exocrine insufficiency (*P* = 0.033) (3). Other authors have reported similar results. Nevertheless, the usage of EN is still controversial owing to its higher prevalence of postoperative pancreatic fistulas and potential risk of malignancy compared with CP.

**Table 1:** Demographic characteristics and outcome evaluations of enucleation.

**References**	**Country of origin**	**Year**	**Study period**	**Type of study**	**SPN (*n*)**	**Enucleation (*n*)**
Wang et al. (3)	China	2018	2009–2016	Retrospective	110	31
Chua et al. (7)	Australia	2016	2000–2015	Meta-analysis	–	1101
Zhou et al. (9)	China	2016	1990–2016	Systematic review	–	1316
Faitot et al. (10)	USA	2015	1998–2011	Retrospective	1	126
Song et al. (11)	Korea	2015	2005–2013	Retrospective	3	65
Wolk et al. (12)	Germany	2015	1996–2013	Retrospective	–	17
Zhang et al. (13)	China	2013	2005–2011	Retrospective	10	119
Cauley et al. (14)	USA	2012	1998–2010	Retrospective	4	45

In this context, the exploration of short- and long-term consequences of EN and its oncologic result is needed. Since most of the existing studies involved small numbers of cases or multicenter series, a meta-analysis makes the interpretation and generalization of findings difficult. Therefore, the purpose of this study was to describe the indications for EN based on our experience, taking into account postoperative outcomes and long-term and oncologic results, as a single-center series study involving a large number of cases.

## Patients and Methods

Clinical data from October 1992 to April 2018, of patients with SPNs who had undergone surgical resection at the Seoul Asan Medical Center, were retrospectively reviewed. The demographic, preoperative, and outcome data (viz., operative procedure, operative time, blood loss, postoperative complications, mortality, time to full feeding, duration of hospital stay, re-operation rate, and recurrence rate) of the patients were analyzed through medical records. This study attempted to objectively compare the tumor size among the patients by measuring the tumor size/intraperitoneal width ratio. We measured the width of the abdominal cavity at the level of the largest point of each tumor in the computed tomography and calculated relative figures for the standardization of tumor sizes by the size of the abdominal cavity (15). The patient's method of operation was determined by considering the size, main pancreatic duct, and location of the mass. Among the complications, pancreatic fistulas (POPFs) were classified into Grades A, B, and C according to the International Study Group (ISGPF) definitions (16). The definition of the malignancy of SPN has lacked consensus to date. In 2000, the World Health Organization (WHO) classified SPN as a borderline neoplasm and solid pseudopapillary carcinoma of the pancreas, characterized by perineural invasion, angioinvasion, or deep infiltration of the surrounding acinar tissue. In 2010, WHO re-defined SPN as a low-grade malignant neoplasm, because the existing perineural invasion, angioinvasion, or deep infiltration of the surrounding acinar tissue do not cause malignant behavior (3). However, there are still no accurate preoperative criteria for the diagnosis of malignant SPN, and in practice, confusion about the malignant criteria still exists (17). This present study classified the SPN stage according to the 2000 WHO classification, to directly reflect the progression status of the disease. The statistical analyses included the Mann-Whitney U test, chi-squared test, and linear by linear association. All analyses were performed with SPSS 21.0 software. Significance was defined at *P* ≤ 0.05.

## Results

Over the course of 27 years, 66 patients underwent SPN surgery at Seoul Asan Medical Center. All patients were pathologically confirmed with SPN. Among them, 15 patients underwent EN surgery (22.7%) and 51 patients underwent CP surgery (77.3%). Of the CPs performed, 8 were a distal pancreatectomy, 22 were a spleen-sparing distal pancreatectomy, 4 were a central pancreatectomy, 16 were a pancreaticoduodenectomy, and 1 was a total pancreatectomy. The demographic characteristics of these 66 patients are shown in Table 2. The mean age of the patients was 14.5 (±5.8) years. The majority of the patients were females (84.8% females vs. 15.1% males). Twenty six patients had the lesion in the head, whereas 40 patients had the lesion in the body and tail region. Three patients with head lesion in the EN group were applied to a preoperative pancreatic stent. One of them underwent open conversion to pylorus-preserving pancreaticoduodenectomy and the other 2 underwent stent removal in 7 days after the surgery. For the entire patient cohort, the average size of the tumor mass was 6.1 cm (±3.3). With regard to the tumor size/intraperitoneal width ratio that was introduced to exclude the relative size of the mass, the total average ratio was 0.26 (±0.13). There was no difference in these ratios between the two surgery groups (EN 0.30 ± 0.1 vs. CP 0.25 ± 0.1, *P* = 0.21).

**Table 2:** Characteristics of the patients in this study.

**Features**	**Total**	**EN group** **(*N* = 15)**	**CP group** **(*N* = 51)**	***P*-value**
Male:Female	10:56	1:14	9:42	0.30
Age (years)	14.5 ± 5.8	14.6 ± 10.7	14.5 ± 3.4	0.96
Body weight (kg)	48.7 ± 13.8	44.7 ± 9.0	49.9 ± 14.9	0.22
Head:Body&tail	26:40	9:6	17:34	0.06
Tumor size (cm)	6.1 ± 3.3	6.10 ± 2.9	6.10 ± 3.5	0.97
T/I width	0.26 ± 0.13	0.30 ± 0.1	0.25 ± 0.1	0.21

*T/I width, ratio of the tumor size to the intraperitoneal width; EN, enucleation; CP, conventional pancreatectomy*.

There was no difference in surgery duration according to the surgery groups (Table 3). Likewise, blood transfusions due to bleeding during the intraoperative duration did not yield significant differences. The difference in the number of patients with margin involvement in the postoperative pathology examination results showed statistical significance (EN 3 vs. CP 2, *P* = 0.04). A total of eight patients (12%) had a malignant tumor, with no statistical difference according to the surgery groups (EN 3 vs. CP 5, *P* = 0.29). Three patients had tumor recurrence (EN 1 vs. CP 2, *P* = 0.54) (Table 4). One of these patients underwent laparoscopic EN for 3 cm mass of tail. There was involvement of resection margin in the permanent biopsy and recurrence occurred in the cut surface at 25 months after surgery. One of two in the CP group received distal pancreatectomy for 13 cm mass of tail, malignant SPN, and multiple lesions recurred in liver after 82 months of surgery. The other one in the CP group received pylorus-preserving pancreaticoduodenectomy for 8.3 cm mass of head, and the aortocaval node recurred after 47 months of surgery.

**Table 3:** Perioperative findings.

	**EN group**	**CP group**	***P*-value**
Duration of operation (min)	262 ± 145	345 ± 195	0.13
Transfusion	1	5	0.71
Margin involvement	3	2	0.04
Malignancy	3	5	0.29

*EN, enucleation; CP, conventional pancreatectomy*.

**Table 4:** Follow-up period, development of diabetes mellitus, and recurrences.

	**Total**	**EN group**	**CP group**	***P*-value**
Follow-up period (months)	511.2	746.8 ± 198.8	442.4 ± 80.1	0.24
Postpancreatectomy DM		0	2 (3.9%)	1.00
HbA1c (%)		5.36 ± 0.93^*^	5.85 ± 0.36^*^	0.30
Recurrence		1 (6.7%)	2 (3.9%)	0.34

DM, diabetes mellitus; EN, enucleation; CP, conventional pancreatectomy

* *EN group (N = 5), CP group (N = 26)*.

Postoperative complications occurred in 42 (63.6%) of the total patient cohort, and there were no mortalities (Table 5). The most frequent complication was pancreatic fistulas: 10 (66.7%) for EN and 32 (62.7%) for CP (*P* = 0.78). The CP group mainly had mild Grade A symptoms (30/32, 93.7%), whereas the EN group had Grades B and C symptoms (8/10, 80%). The incidence of Grades B and C POPFs was different between the two groups (*P* < 0.001). Consequently, the duration of maintaining drainage with POPF was longer in the EN group, which held statistical significance (EN 21.2 ± 4.5 days vs. CP 12.6 ± 1.9 days, *P* = 0.026).

**Table 5:** Short-term outcomes.

	**EN group**	**CP group**	***P*-value**
Fluid collection	1	9	0.43
Splenic infarction	0	4	0.26
Hematoma	0	1	0.58
Pancreatic fistula	10 (66.7%)	32 (62.7%)	0.78
Grade A	2	30	0.002
Grades B & C	8	2	<0.001
PV thrombosis	0	1	0.59
Chylous drainage	3	1	0.034
Duration with drainage (days)	21.2 ± 4.5	12.6 ± 1.9	0.026
Time to oral intake (days)	17.0 ± 8.7	5.1 ± 3.3	<0.001
Time to full feeding (days)	19.9 ± 9.4	7.7 ± 3.9	<0.001
Hospital stay (days)	23.4 ± 10.0	13.2 ± 6.5	0.002

*PV, portal vein; EN, enucleation; CP, conventional pancreatectomy*.

Complications other than POPFs were fluid collection, splenic infarctions, hematomas, portal vein thromboses, and chylous drainage. There were no serious cases requiring surgery or invasive procedures. The incidences of these complications were not different between the groups. Patients in the EN group took more time to reach full feeding (EN 19.9 ± 9.4 days vs. CP 7.7 ± 3.9 days, *P* < 0.001) and had longer hospital stays (EN 23.4 ± 10.0 days vs. CP 13.2 ± 6.5 days, *P* = 0.002).

With regard to postoperative long-term outcomes, 62 patients were available for the follow-up evaluation, whereas the other 4 patients had not visited the institution for more than 1 year. The average follow-up period was 511.2 months for the entire patient cohort: 746.8 months (range, 10–2280 months) for the EN group and 442.4 months (range, 15–2127 months) for the CP group. The results regarding diabetes are presented in Table 4. Two patients (3.9%) developed DM after surgery, both of whom had undergone distal pancreatectomy. There was no significant difference in the post-pancreatectomy HbA1c examination results for the two groups (EN 5.36 ± 0.93%vs. CP 5.85 ± 0.36, *P* = 0.30). However, the HbA1c test was not conducted for all the patients, and the small number of samples did not allow reliable calculation of statistical significance (EN 5 patients vs. CP 26 patients).

With regard to the oncologic results, there were three recurrences (EN 1/15 (6.7%) vs. CP 2/51 (3.9%), *P* = 0.34). One of the three patients had undergone laparoscopic EN with resection margin involvement found in the biopsy specimen; the site of the recurred mass was also in the resection margin. The patients with tumor recurrences were all re-operated on by complete resection.

## Discussion

SPN, a low-grade malignant neoplasm that has a low mortality rate, is resected using standard operative techniques, such as pancreaticoduodenectomy and distal pancreatectomy, after the complete resection. Although the rates of morbidity and mortality are reduced by the procedure, the incidence of postoperative endocrine and exocrine pancreatic insufficiencies is still a matter of concern when considering the use of complete resection for infant treatment. As an alternative treatment, pancreatic EN can save the normal pancreatic parenchyma and decrease the risk of endocrine and exocrine insufficiencies (3, 5–7, 14). Moreover, compared with CP, EN is technically simpler, and its shorter surgery duration and less blood loss have been frequently mentioned in previous studies (9, 11, 14), which is consistent with our findings.

Therefore, owing to the need for parenchymal preservation, consideration of the need for complete resection is required before surgery, and the application of surgical methods is restricted. Previous studies have reported that the recurrence rate of SPN ranges from 4.4 to 20%, and the death rate from this disease was 0.9–7% (4, 18). In this study, three patients in the EN group had resection margin involvement. Among them, one patient had a recurrence at 2 years after the first surgery. With regard to the oncologic aspect, it would be better to check the operative margin by conducting a frozen biopsy in the operating room (11).

**Figure 1:**
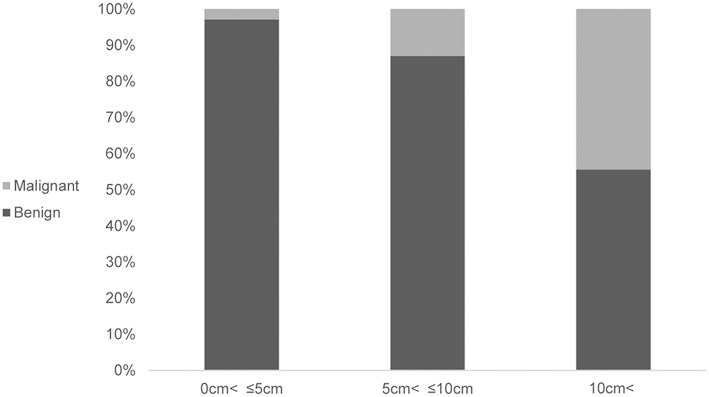
Association between the tumor size and malignancy.

Furthermore, securing a resection margin is more important in the case of a malignant SPN. If the intraoperative frozen biopsy of the enucleated lesion shows an invasive malignancy, then the conversion to conventional resection should be considered. Therefore, when performing imaging evaluation before surgery, evaluation of the malignancy is needed for the consideration of a complete resection. A large tumor size (>8 cm), WHO criteria of a pseudopapillary cancer (i.e., perineural invasion, angioinvasion, and adjacent organ invasion), and Stage IV tumors were significantly associated with recurrent pancreatic SPNs (19). In this analysis, an association between the tumor size and malignancy was identified (*P* = 0.002) (Figure 1). In the case of a presumed malignant SPN from the imaging evaluation before surgery, EN cannot be recommended.

The mortality (0% in both groups) and morbidity (EN 93.3% vs. CP 94.1%) rates among the patients who underwent EN and CP were similar. The morbidity of patients in the EN group (93.3%) was high in our patient cohort. Nevertheless, it is common for more than half of the patients to have complications after pancreatic surgery (12–14). Based on the stricter Clavien–Dindo classification of major complications (where Grades IIIb to V are major complications), those in this study were lower at 6.7% (1/15) (20). Wolk et al. reported an overall morbidity of 82.4%, with 0% of major complications after EN. Compared with major pancreatic resections, the overall morbidity rates were comparable with EN (80.8 vs. 82.4%) (12). Other authors have also observed ~60–70% of morbidity and ~5–13% incidences of major complications (13, 14).

The most frequent EN complication in this study was POPF (66.7%), which was similar to that of other published studies (3, 9–11, 13, 14, 21). The patients in the CP group had a high rate of Grade A (highly light) POPF [30/32 (93.7%)], whereas those in the EN group mainly had Grade B POPF [8/10 (80.0%)]. The Grade B POPF led to longer periods of NPO and hospitalization of the patients in the EN group (*P* < 0.001 and *P* = 0.002, respectively). Some studies have shown that a short distance (<2–3 mm) between the tumor and the main pancreatic duct is a risk factor for the development of pancreatic fistulas (9–13, 22). Other studies have suggested preoperative endoscopic pancreatic stent placement as an intraoperative guide to prevent damage of the main pancreatic duct (11). In this study, a preoperative pancreatic stent was applied in the case of a high proximity between the mass and main pancreatic duct. As one patient turned out to have main pancreatic duct injury during surgery from the stent check, the conversion to pylorus-preserving pancreaticoduodenectomy was performed. Although there was no difference in POPF risks according to location in this study, Faitot et al. observed that EN for a tumor located in the head/uncus was the only independent predictive factor for POPF. Thus, the ductal injury according to tumor location should be taken into consideration (10). Although this technique can contribute to the prevention of main pancreatic duct injury, no persuasive evidence has as yet been suggested in the literature.

The advantage of EN over CP is that it can maintain endocrine/exocrine function by saving the normal pancreatic parenchyma. Pancreatic function is an important factor for quality of life, especially for infants. The incidences of endocrine and exocrine insufficiencies would lead to a life-time of replacement treatments. Previous reports have documented that the pancreatic exocrine and endocrine functions can be well-preserved by EN, and that the procedure is superior to CP (5, 7, 9, 10, 14). Nevertheless, the association between the remnant pancreas volume and endocrine function remains controversial. Some studies have indicated that the incidence of postpancreatectomy DM was low, or that there was no association between the residual pancreas volume and pancreatogenic DM (23, 24). On the other hand, several studies have reported that the pancreatic resection volume is a risk factor for postpancreatectomy DM. Falconi et al. showed a 3% incidence of endocrine insufficiency for a pancreatic parenchymal-preserving resection compared with the rates of 18% for a pancreaticoduodenectomy and 14% for a distal pancreatectomy (25). Similarly, Kwon et al. found that the resection volume of the pancreas was associated with pancreatectomy-induced DM after a distal pancreatectomy, particularly in patients with a resected pancreatic volume rate larger than 35.6% (6). This study also showed that the EN group had a lower rate of postoperative endocrine insufficiency (including DM and abnormal glucose homeostasis), and postpancreatectomy DM occurred only in the patients who had undergone a distal pancreatectomy (*P* = 0.44 and *P* = 0.30, respectively).

In conclusion, EN of SPNs should be selectively applied under a few conditions. First, in the case of a tumor size of <5 cm, imaging evaluation before surgery might rule out a malignant SPN. In the case of a tumor size of >5 cm, the aspects that follow might additionally be taken into consideration because of the relatively high malignant potential. Second, the main pancreatic duct should not be involved. Third, the location of the tumor mass should be considered. In the case of a mass located distally, a pancreas-preserving procedure is recommended if the resection volume is larger than 35%, i.e., approximately mass involving more than 2/3 of the tail. Based on such selection criteria, EN might be useful as a safe procedure for SPN treatment in children, with its advantages of preventing tumor recurrence and decreasing the POPF incidence.

## Data Availability

All datasets generated for this study are included in the manuscript and/or the supplementary files.

## Ethics Statement

All procedures performed in studies involving human participants were in accordance with the ethical standards of the ethic committee of Asan Medical Center Children's Hospital of Ulsan University (IRB No. S2018-2030-0001).

## Author Contributions

All authors listed have made a substantial, direct and intellectual contribution to the work, and approved it for publication.

### Conflict of Interest Statement

The authors declare that the resear.ch was conducted in the absence of any commercial or financial relationships that could be construed as a potential conflict of interest.
